# Comparative analysis of diagnostic colonoscopy in symptomatic young adults from South Korea and the United States

**DOI:** 10.1097/MD.0000000000007504

**Published:** 2017-09-01

**Authors:** Min Seob Kwak, Jae Myung Cha, Jeong-Sik Byeon, Otto S. Lin, Richard A. Kozarek

**Affiliations:** a Department of Internal Medicine, Kyung Hee University Hospital at Gangdong, Kyung Hee University College of Medicine; b Department of Gastroenterology, University of Ulsan College of Medicine, Asan Medical Center, Seoul, Korea; c Digestive Disease Institute, Virginia Mason Medical Center, Seattle, WA.

**Keywords:** colon, colonoscopy, ethnic difference, neoplasm, symptom

## Abstract

To date, not much is known about ethnic differences in the prevalence of colorectal neoplasia in symptomatic young patients with lower gastrointestinal symptoms. This study sought to compare diagnostic colonoscopic findings in symptomatic young patients from South Korea and the United States. Results from the first diagnostic colonoscopies in symptomatic 18- to 49-year-old patients were compared between the United States and Korean cohorts. The US cohort data were collected at Virginia Mason Medical Center in Seattle, Washington between January 2007 and January 2010, and the Korean cohort data were collected at 14 university hospitals in Korea between June 2006 and June 2015.

The prevalence of advanced neoplasias was similar in both cohorts for bleeding and nonbleeding symptoms (*P* = .966 and *P* = .076, respectively). In a subgroup analysis for 40- to 49-year-old patients, the prevalence of advanced neoplasias was similar for bleeding symptoms; however, nonbleeding symptoms were significantly higher in the Korean cohort than in the US cohort (6.2% vs 2.6%, *P* < .001). In an age subgroup analysis for 18- to 39-year-old patients, the prevalence of advanced neoplasias was similar for bleeding and nonbleeding symptoms in both cohorts. Multivariate analysis showed that lower gastrointestinal symptoms were not associated with the risk of any type of advanced neoplasia in young Korean patients.

Ethnic disparities in the prevalence of advanced neoplasia on diagnostic colonoscopy were not noticeable between Korean and US young patients. However, 40- to 49-year-old patients with nonbleeding symptoms require more attention to detect advanced neoplasia in Korea than similarly aged patients in the United States.

## 1 Introduction

One of the major roles of diagnostic colonoscopy in patients with a variety of lower gastrointestinal (LGI) symptoms is to detect advanced colorectal neoplasia, even though benign causes are much more common.^[1–3]^ To date, weighing the potential benefits of diagnostic colonoscopy against the expected low yields of advanced neoplasia in symptomatic young adults is difficult because little information on this issue is available. Although the concept of “alarm” symptoms has been proposed to weigh the potential benefits of colonoscopy against the expected low yields for advanced neoplasia in symptomatic patients,^[4]^ the threshold for diagnostic colonoscopy to detect advanced neoplasia must be individualized in symptomatic young adults.^[5]^ Currently, little is known about ethnic disparities in the prevalence of colorectal neoplasia on diagnostic colonoscopy (especially in young patients) aside from knowledge already gained by screening colonoscopy.^[6,7]^

In our previous study,^[5]^ rectal bleeding warranted diagnostic colonoscopy to detect advanced neoplasia, whereas nonbleeding symptoms were associated with a lower risk of advanced neoplasia. In previous studies focused on young patients with rectal bleeding,^[8–10]^ a 2.4% cancer prevalence in a Pakistani study^[8]^ was contrasted with a 0.0% to 0.03% cancer prevalence in American studies.^[9,10]^ These results suggest ethnic disparities in the prevalence of advanced neoplasia on diagnostic colonoscopy in young patients with rectal bleeding. However, all of these studies were limited due to small sample sizes and a focus only on rectal bleeding.^[8–10]^ Therefore, ethnic disparities in the prevalence of advanced neoplasia should be evaluated for all LGI symptoms on diagnostic colonoscopy in young patients. Furthermore, colorectal cancer (CRC) in young patients is increasing^[7,11–13]^ and has a more aggressive disease course than in older patients.^[14,15]^

In the present study, we compared the prevalence of colorectal neoplasia in symptomatic young patients (aged 18–49 years) in the US and Korean cohorts and analyzed patients according to various LGI symptoms.

## 2 Patients and methods

### 2.1 Patients and study design

The results of first diagnostic colonoscopies for symptomatic young (aged 18–49 years) patients with LGI symptoms were compared between US and South Korean patients. For the US cohort, the database of diagnostic colonoscopy for symptomatic American patients aged 18 to 49 years from Virginia Mason Medical Center in Seattle, Washington, between January 2007 and January 2010, was used, as described previously.^[5]^ For the Korean cohort, data from diagnostic colonoscopy for symptomatic Korean patients aged 18 to 49 years were collected at 14 university hospitals in Korea between June 2006 and June 2015. For both cohorts, potential subjects were excluded if they met any of the following criteria: previous history of colonoscopy or sigmoidoscopy, incomplete colonoscopy due to poor bowel preparation or failure to achieve cecal intubation, or personal history of colorectal neoplasia, inflammatory bowel disease, or genetic syndromes. To avoid potential bias by patients with genetic syndromes, patients with a strong family history of CRC (ie, a history of CRC in family members <60 years or multiple family histories in first-degree relatives) were also excluded from the analysis. Physicians (in the Korean cohort) or a specially trained, nonphysician research nurse (in the US cohort) reviewed the electronic medical records of each subject for demographics, colonoscopy findings, and pathology reports in both cohorts. This study was approved by the institutional review board of each participating hospital (KHNMC 2015-07-004 and VMMC IRB 30103000). Because this was a retrospective analysis of data previously collected, the need for individual informed consent was waived.

### 2.2 Definition of indications

For both cohorts, the indications of colonoscopy were grouped according to the same definitions, following the appropriate criteria recommended in the United States and Europe.^[16,17]^ We defined 6 symptom categories, as described previously^[5]^: anemia (low hematocrit without occult bleeding or hematochezia), rectal bleeding (hematochezia or other visible forms of bleeding), occult bleeding (nonvisible bleeding detectable only by fecal occult blood tests), unintentional weight loss (>10% of baseline weight), changes in bowel habits or new-onset or markedly worsened constipation or diarrhea, and abdominal pain or discomfort. Bleeding symptoms were defined as anemia, rectal bleeding, or occult bleeding, whereas nonbleeding symptoms were defined as a change in bowel habit, significant unintentional weight loss, or abdominal pain or discomfort.

### 2.3 Endoscopic procedures

In both cohorts, all colonoscopies were performed by experienced gastroenterology staff or fellows using Olympus CF endoscopes (Olympus, Tokyo, Japan) after bowel preparation with a split-dose regimen. All detected polyps were photographed, and their characteristics (location, size, and shape) were documented. The proximal colon was defined as all areas proximal to the splenic flexure, whereas the distal colon included all areas distal to and including the splenic flexure. Gastrointestinal pathologists of each respective hospital performed histological evaluation for all excised polyps. Advanced neoplasms were defined as any advanced adenoma (ie, adenoma with a diameter ≥10 mm, high-grade dysplasia, or >25% villous features) or carcinoma.^[18]^ In patients with multiple neoplasms, the neoplasm with the most advanced pathology or the largest size was reported. The findings obtained during colonoscopy were classified into: no neoplasia, any neoplasia, or advanced neoplasia. In this study, only advanced neoplasia was regarded as a clinically relevant finding of diagnostic colonoscopy, as this is suggested to be the most appropriate target for colonoscopy, and early detection of these lesions can improve cancer-related survival.^[19–24]^

### 2.4 Data analysis

The primary endpoint was the prevalence of advanced neoplasia in the 2 cohorts. Secondary endpoints included the prevalence of adenomas in the 2 cohorts. The *χ*^2^ test or Fisher exact test was used to compare proportions, and Student *t* test or nonparametric Mann-Whitney *U* test were used to compare means. All *P* values were 2-tailed, and a *P* value <.05 was considered statistically significant. Statistical analyses were performed using the Statistical Package for the Social Sciences version 18.0 for Windows (SPSS Inc, Chicago, IL).

## 3 Results

During the study period, 1266 and 1964 symptomatic young patients were included in the US and South Korean cohorts, respectively. The colonoscopy completion rate was >97.0% in both cohorts.

### 3.1 US and Korean cohorts

The demographic and baseline data for the study populations are presented in Table 1. Median ages in the US and Korean cohorts were 40.4 years [standard deviation (SD), 8.0] and 42.5 years (SD, 5.2), respectively. For the indications of colonoscopy, occult bleeding and unintentional weight loss were similar in the 2 cohorts. However, rectal bleeding and bowel habit changes were more frequent indications of diagnostic colonoscopy in US patients than in Korean patients (both *P* < .001). Conversely, anemia and abdominal pain were more frequent indications of diagnostic colonoscopy in Korean patients than in US patients (*P* = .046 and *P* < .001, respectively).

**Table 1:**
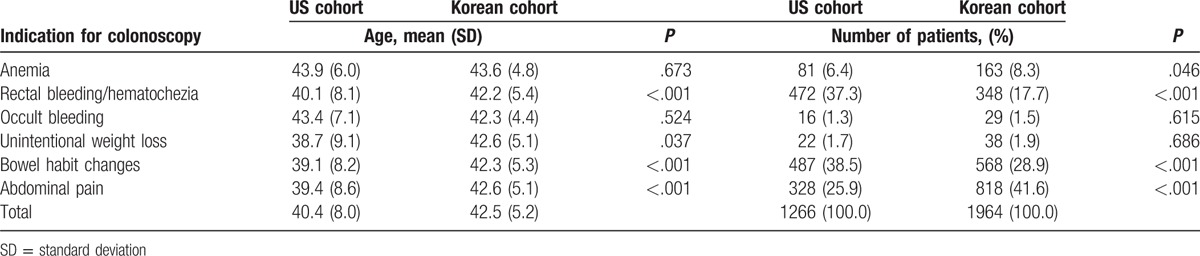
Baseline demographic characteristics of US and Korean cohorts.

### 3.2 Prevalence of colorectal neoplasia

The prevalence of any neoplasia and advanced neoplasia was compared between the Korean and US cohorts according to bleeding and nonbleeding symptoms (Table 2). The prevalence of advanced neoplasia was similar in the 2 cohorts for bleeding and nonbleeding symptoms (5.7% vs 5.8%, *P* = .966 and 4.8% vs 3.2%, *P* = .076, respectively). The prevalence of any neoplasia was similar in the 2 cohorts for bleeding symptoms (22.4% vs 21.8%, *P* = .805), but it was significantly higher in the Korean cohort than in the US cohort for nonbleeding symptoms (23.6% vs 14.3%, *P* < .001).

**Table 2:**
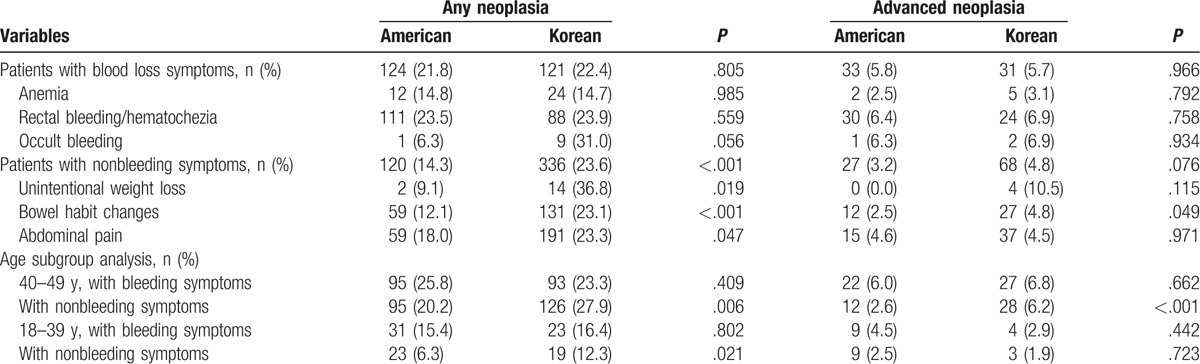
Prevalence of colorectal neoplasia in US and Korean cohorts.

In an age subgroup analysis of patients aged 40 to 49 years, the prevalence of advanced neoplasia was similar for bleeding symptoms, but nonbleeding symptom prevalence was significantly higher in the Korean cohort than in the US cohort (6.2% vs 2.6%, *P* < .001). In patients aged 18 to 39 years, the prevalence of advanced neoplasia was similar for bleeding symptoms and nonbleeding symptoms in both cohorts. The prevalence of any neoplasia was similar for bleeding symptoms, but it was significantly higher in the Korean cohort than in the US cohort for nonbleeding symptoms in both the younger (aged 18–39 years) and the older (aged 40–49 years) patient groups.

### 3.3 Location of colorectal neoplasia

The prevalence of advanced neoplasia was stratified by anatomic location of tumor(s) for each LGI symptom (Table 3). Advanced neoplasia was more prevalent in the distal colon than the proximal colon in both cohorts. However, the prevalence of advanced neoplasia stratified by anatomic location of tumor(s) was not significantly different between the cohorts.

**Table 3:**
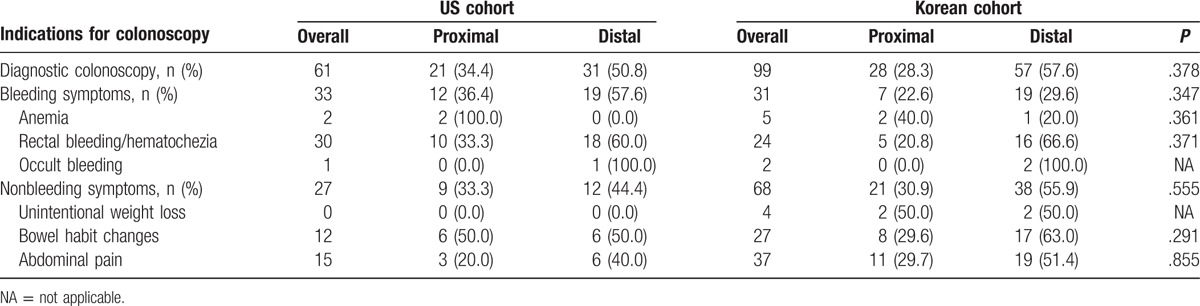
Prevalence of advanced neoplasia, stratified by anatomic location of tumor.

### 3.4 Predictive factors for colorectal neoplasia in the Korean cohort

Predictive factors related to colorectal neoplasia in the Korean cohort were investigated by multivariate analysis (Table 4). Age and male sex were significantly associated with the risk of any neoplasia or advanced neoplasia in the young Korean cohort. However, no LGI symptoms were associated with the risk of any neoplasia or advanced neoplasia.

**Table 4:**
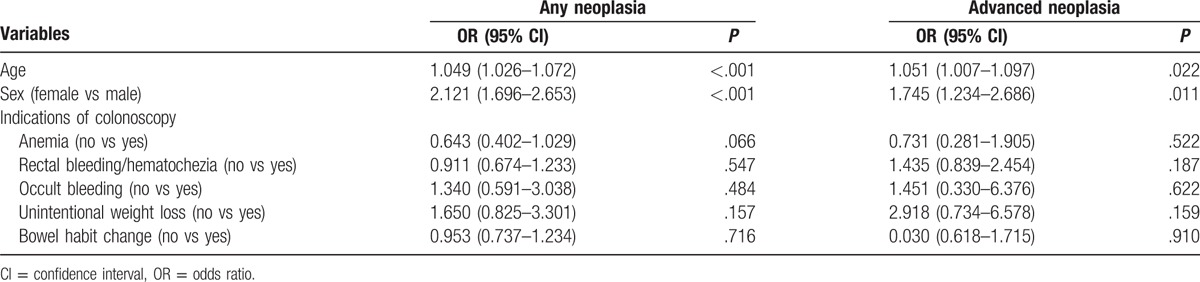
Multivariate analyses of colorectal neoplasia in the Korean cohort.

## 4 Discussion

Until now, little has been known about ethnic disparities in the prevalence of colorectal neoplasia on diagnostic colonoscopy, especially in young populations. This study showed that the prevalence of advanced neoplasia on diagnostic colonoscopy was not significantly different between US and Korean cohorts, regardless of bleeding or nonbleeding symptoms. Our data also showed that, in a subgroup analysis (patients aged 40–49 years), the prevalence of advanced neoplasia was similar in the 2 cohorts for patients with bleeding symptoms. However, it was significantly higher in the Korean cohort than in the US cohort for patients with nonbleeding symptoms. In a different subgroup (patients aged 18–39), the prevalence of advanced neoplasia was similar for patients with bleeding symptoms or nonbleeding symptoms in the 2 cohorts. To summarize, ethnic disparities in the prevalence of advanced neoplasia on diagnostic colonoscopy were not noticeable between young Korean and US patients. However, patients aged 40 to 49 years with nonbleeding symptoms should receive increased scrutiny for detecting advanced neoplasia in Korea, as these patients had a higher prevalence of advanced neoplasia.

Among LGI symptoms, rectal bleeding, abdominal pain, and bowel habit changes were the most common symptoms of CRC.^[25,26]^ In a previous study that focused on diagnostic colonoscopy,^[27]^ the benefit of colonoscopy was greatest in the presence of bleeding symptoms, such as rectal bleeding or occult bleeding. In contrast, previous studies on diagnostic colonoscopy in patients with nonbleeding symptoms mostly have shown low yields for advanced neoplasia.^[28,29]^ In a US study,^[30]^ however, patients with persistent nonbleeding symptoms (including abdominal pain or bowel habit changes) had almost as high a yield of colorectal neoplasia as those with rectal bleeding, consistent with our findings. An Asian consensus on irritable bowel syndrome recommended that all patients presenting with recurrent abdominal pain of 3 months or longer duration should be screened for malignancy.^[31]^ In this regard, abdominal pain has been the most important reason for gastrointestinal consultation in Asian patients with irritable bowel syndrome.^[32–34]^ Similarly, in our Korean cohort, abdominal pain was more frequently an indication for colonoscopy than the traditional alarm symptoms, such as bowel habit change or unintended weight loss.^[35]^ Considering the similar yields for advanced colorectal neoplasia in patients with abdominal pain and patients with other LGI symptoms in our study, the previous emphasis on abdominal pain as a risk factor for malignancy may be exaggerated.

Multivariate analyses for colorectal neoplasia in the Korean cohort showed age and male sex were significantly associated with risk of colorectal neoplasia, while no LGI symptoms were associated with risk of colorectal neoplasia. To summarize, the presence of LGI symptoms, including those traditionally regarded as alarm symptoms for CRC, was not associated with risk for colorectal neoplasia. Rather, old age and male sex have been established as well-known risk factors for colorectal neoplasia in previous studies.^[32,33,35]^ Considering the higher risk of colorectal neoplasia on diagnostic as well as on screening colonoscopy in men,^[20,30,34,36]^ the threshold for a diagnostic colonoscopy to detect colorectal neoplasia may be lower in men than in women, similar to the case for screening colonoscopy.^[36]^

The present study has several strengths. First, this is the first description of a ethnic disparity in the prevalence of colorectal neoplasia between Korean and US cohorts according to various LGI symptoms. Thus, the present study may add new information about ethnic disparity on diagnostic colonoscopy as well as on screening colonoscopy. Second, this study included a large amount of diagnostic colonoscopy data from the US and Korea. The total numbers of subjects in these analyses were sufficient to detect a difference between the Korean and US populations. Third, 1 physician (J.M.C.) reviewed the database of diagnostic colonoscopy in both cohorts, using the same definitions of symptom variables. This was done to minimize the influence of selection bias. However, there are several limitations that warrant consideration. First, some confounding factors from the unequal distribution of the symptoms for diagnostic colonoscopy between the 2 cohorts may exist. Nevertheless, the unequal distribution of the symptoms between the 2 cohorts may reflect the real indications of diagnostic colonoscopy in both countries. Second, LGI symptoms reported by patients are often vague, and it is difficult for physicians to reliably distinguish those symptoms. As mentioned, this bias was minimized by having one physician review all databases, using the same definitions in both cohorts. Third, there is a possible selection bias, as differences in the medical reimbursement systems of both countries may have affected the composition of the cohorts. Therefore, prospective, large-scale studies adjusting for various confounding factors are warranted to address this issue.

In conclusion, ethnic disparities in the prevalence of advanced neoplasia on diagnostic colonoscopy were not noticeable between young Korean and US patients. However, patients aged 40 to 49 with nonbleeding symptoms should receive increased scrutiny for detecting advanced neoplasia in Korea.

## Acknowledgments

The authors would like to thank Hyun Gun Kim, Young-Seok Cho, Jeong Eun Shin, Kyeong Ok Kim, Hyo-Joon Yang, Hoon Sup Koo, Young-Eun Joo, Sun-Jin Boo, Yunho Jung, Jun Lee, Hyun Jung Lee, Jongha Park, and Chang Mo Moon for their contributions to data acquisition in the Korean cohort. All authors are members of the Intestinal Cancer Study Group of the Korean Association for the Study of Intestinal Diseases (KASID).
